# Pigeon Pea and Cowpea-Based Cropping Systems Improve Vesicular Arbuscular Mycorrhizal Fungal Colonisation of Subsequent Maize on the Alfisols in Central Malawi

**DOI:** 10.1155/2017/2096314

**Published:** 2017-05-11

**Authors:** Keston O. W. Njira, Ernest Semu, Jerome P. Mrema, Patson C. Nalivata

**Affiliations:** ^1^Department of Soil and Geological Sciences, Sokoine University of Agriculture, P.O. Box 3008, Morogoro, Tanzania; ^2^Department of Crop and Soil Sciences, Lilongwe University of Agriculture and Natural Resources, P.O. Box 219, Lilongwe, Malawi

## Abstract

Mycorrhizal associations contribute to the sustainability of crop production systems through their roles in nutrient cycling and other benefits in the soil-plant ecosystems. A two-year study was conducted on the Alfisols of Lilongwe and Dowa districts, Central Malawi, to assess the vesicular-arbuscular mycorrhizal (VAM) fungal colonisation levels in pigeon pea, cowpea, and maize grown in sole cropping, legume-cereal, and legume-legume intercropping systems and in the maize grown in short rotation (year 2) as influenced by the previous cropping systems and N fertilizer application. The gridline intersect method was used to assess the VAM fungal colonisation levels. Results showed that all treatments that included legumes whether grown as sole crop, in legume-cereal or in legume-legume cropping systems in the previous year, had significantly higher (*P* < 0.05) VAM fungal colonisation of the rotational maize crop roots by a range 39% to 50% and 19% to 47% than those in maize supplied and not supplied with N fertilizer, respectively, in a maize-maize short rotation, at the Lilongwe site. A similar trend was reported for the Dowa site. Furthermore, there were positive correlations between VAM fungal colonisation and the plant P content, dry matter yield, and nodule numbers. Further studies may help to assess the diversity of VAM fungal species in Malawi soils and identify more adaptive ones for inoculation studies.

## 1. Introduction

The sustainable intensification of crop production calls for various approaches including integrated soil fertility management (ISFM) [1, 2]. It advocates agricultural productivity while ensuring the maintenance and resilience of the ecosystems [3, 4]. On other hand, soil fertility decline is one of the main challenges that can continue to derail sustainable agriculture production in many developing countries. In sub-Saharan Africa, most smallholder farmers have limited capabilities to acquire inorganic fertilizers [5]. Inclusion of legumes that improves soil fertility through biological nitrogen fixation (BNF) and phosphorus (P) acquisition through mycorrhizal associations is of paramount importance.

Mycorrhiza is a mutualistic association between roots of plants and some fungal species. Major groups of mycorrhizae include ectomycorrhizae, endomycorrhizae, ericoid mycorrhizae, and orchid mycorrhizae [6–8]. Endomycorrhizae involves root cortex penetrating fungi under a phylum of Glomeromycota, associating with over 80% of plant species [6, 9]. The most reported endomycorrhiza group is commonly referred to as vesicular-arbuscular mycorrhiza (VAM) or arbuscular mycorrhiza (AM) because of their morphological features, the arbuscules and vesicles, which are used for transportation and storage of materials, respectively [6, 10]. The mycorrhizal association is developed as a mutualistic adaptation benefiting both symbionts. The fungal species benefits carbohydrates and habitat from the plant while providing a number of benefits to the plant. They enhance P uptake and other nutrients by increasing plant root surface area and producing organic acids and phosphatase enzymes that solubilise P [11–13]. Marschner and Dell [14] have reported up to 80%, 25%, 10%, 25%, and 60% uptake of plant P, N, K, Zn, and Cu, respectively, by external hyphae of VAM. Furthermore, some studies have shown synergistic effects of VAM to* Rhizobium*-legume symbiosis that result in BNF increase [15, 16]. This is achieved through the VAM's enhancement of plant P uptake, which is required in high amounts for BNF [10]. The VAM associations are also reported to be involved in N transfer from legume to cereal in intercropping systems [17]. Other benefits to the plant offered by the mycorrhiza include enhancement of the plant's water uptake that increases drought tolerance [18, 19], increasing the plant's resistance against some soil borne pathogens [20, 21] and against weed species such as striga [22]. Furthermore, mycorrhizal fungi produce glomalin, a glycoprotein that binds soil particles and improves the soil structure [23]. It also binds heavy metals and improves plant's tolerance to their toxic effects [23].

The proliferation of VAM fungi in an ecosystem is affected by a number of factors. Many studies show that VAM development usually favours low levels of P [24] with a few exceptions where additional P increased colonisation levels [25], slightly low pH [26], warm temperatures, and light availability [27]. However, influences of farming practices and cropping systems have shown different outcomes. Conservation practices such as conservation agriculture (CA) have been associated with increased VAM fungal diversity in some studies [28] and rotations with some legumes have shown increased VAM fungal colonisation in the crop that follow [29]. On the other hand intercrops have led to increase while others have led to decrease in either VAM fungal colonisation or diversity [21, 30].

In Malawi, common crops include maize* (Zea mays)*, common beans* (Phaseolus vulgaris)*, cowpea* (Vigna unguiculata)*, pigeon pea* (Cajanus cajan)*, and groundnuts* (Arachis hypogaea)* and some agroforestry species. These crops are usually grown in monocrops/sole crops and in intercrops. Jefwa [30] reported the presence of VAM species of the genera* Glomus*,* Gigaspora*,* Acaulospora*,* Scutellospora*, and* Archaeospora* in a study involving sole cropping and intercropping of maize with agroforestry species of* Gliricidia sepium*,* Sesbania sesban*, and* Sesbania macrantha* on the soils of Southern Malawi. However, despite the importance of VAM in the cropping systems, information on the status of VAM in the field crops such as pigeon pea and cowpea grown in sole crops or intercropped with maize or grown as legume/legume intercrops on the Malawi soils is scanty. Therefore, the aim of this study was to assess VAM fungal colonisation status in the cowpea, pigeon pea, and maize grown as sole crops, legume-cereal, and legume-legume intercrops and on maize grown after the legume-based systems as a short rotation. Furthermore, it was also aimed at assessment of correlations of VAM fungal colonisation in the intercrops and P uptake, BNF, and other yield components.

## 2. Materials and Methods

### 2.1. Site Description

The study was conducted in two cropping seasons (2013/14 and 2014/15) in two sites of Central Malawi, in the districts of Lilongwe and Dowa. The experiment was conducted at the Lilongwe University of Agriculture and Natural Resources, Bunda Campus Research farm (14°11′S, 33°46′E) in the Lilongwe district, whereas in the Dowa district, the experiment was conducted at the Nachisaka Extension Planning Area (EPA) (13°37′S, 33°56′E). The soils at both sites are classified as Alfisols (using the USDA Soil Taxonomy System) or Luvisols (using the World Reference Base System) [32, 33]. Based on critical values as outlined by Chilimba [34] soil analysis results before planting in the 2013/14 cropping season showed very low mean value of total N (0.05%), high available Mehlich-3 P (57 mg kg^−1^), low soil organic matter (1.8%), slightly acid soil reaction (6.0), and sandy clay loam texture in the 0–20 depth range, for the Lilongwe site. For the Dowa site, results of soil analysis indicated medium N (0.14%), moderately high available P (41 mg kg^−1^), high soil organic matter (4.7%), and sandy clay loam texture in the 0–20 depth range. Both areas receive unimodal rainfall from mid-November to early-April. The total rainfall amounts for the Lilongwe site were 1205 mm and 639 mm in the 2013/14 and 2014/15 cropping seasons, respectively. For the Dowa site, the total rainfall amounts were 758 mm and 577 mm in the 2013/14 and 2014/15 cropping seasons, respectively.

### 2.2. Treatment Description

The assessment of VAM fungal colonisation was superimposed on a major experiment where many other variables were tested. The first year involved planting cowpea, pigeon pea, and maize as sole crops, legume/legume, and legume/cereal in-row intercrops. The treatments were replicated three times and were laid out in a randomized complete block design (RCBD) in the first season and split-plot in the second season. The size of the plots for the first season was 15 m by 7 m. In both sole cropping and intercropping treatments, maize and pigeon pea were planted 3 seeds per planting station and 90 cm between planting stations within the row/ridge. This led to a planting pattern where a pigeon pea planting station was systematically in the middle of two maize planting stations. On the other hand, cowpea was planted two seeds per planting station spaced at 20 cm along the row and in the intercropping three planting stations were fitted in between planting stations of maize or pigeon pea. These planting patterns form in-row intercropping systems commonly practiced and recommended in Malawi [35].

The second year (cropping season two) involved testing the residual effects of the different cropping systems on short rotational maize yields by planting maize across all treatment plots. Therefore VAM fungal colonisation was also assessed on the short rotational maize. Each type of crop residue was incorporated in a plot where that specific crop was grown. During VAM colonisation assessment, plots of the previous season were purposively split into two subplots hence a split-plot design was achieved with previous cropping system as main factor and N fertilizer levels of 0 kg N ha^−1^ and 23 kg N ha^−1^ as subfactors. It should be noted that the VAM fungi under this study were not inoculated but only indigenous ones were assessed.

### 2.3. Assessment of VAM Fungal Colonisation

**Figure 1:**
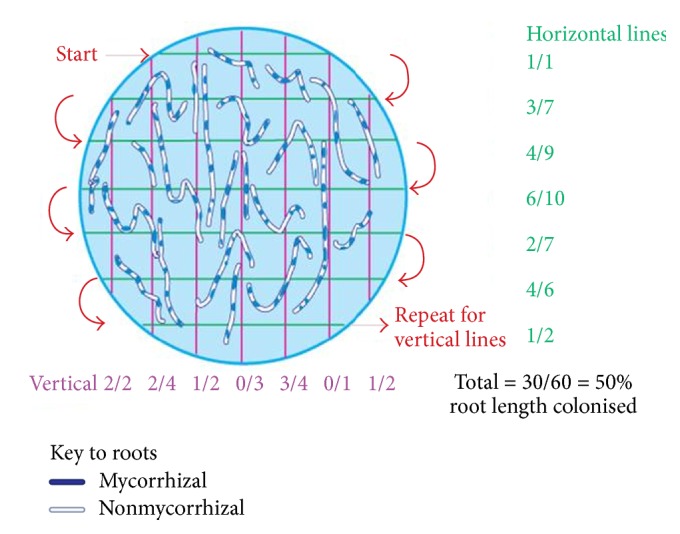
The illustration of how percent root length colonised is determined using the gridline intersect method. Source: Brundrett, [31].* Note*. The diagram shows a grid lined petri dish containing root threads.

The process of assessing VAM fungal colonisation in plant roots involved plant sampling to obtain the roots, clearing of roots of various pigments and staining them to make hyphae and VAM key features, that is, arbuscules and vesicles, visible on a compound light microscope, and quantifying of VAM fungal colonisation of roots on a dissecting microscope. Ten plants were sampled per plot of each treatment. Roots were cut from stems of the uprooted plants and gently cleaned, placed in clean plastic bottles, and transported while being kept in a cooler box. Ethanol was added to the bottles when keeping them in a refrigerator. The clearing and staining of roots were done using a procedure as described by Vierheiling et al. [36] and Cao et al. [37] in which 10% potassium hydroxide (KOH) is used for clearing root pigments at 90°C for 90 min, blanching with alkaline 10% hydrogen peroxide (H_2_O_2_), acidifying with 0.2 M hydrochloric acid (HCl), and staining with 5% blue ink in 5% acetic acid (vinegar). Verification of VAM presence was done on a compound microscope (10 × 40 magnification) by considering features as described by Brundrett [38]. Quantification of percent root length colonisation was done using the gridline intersect method as described by Giovannetti and Mosse [39].  Figure 1 shows how the counts of root colonisation were done as illustrated by Brundrett [31].

### 2.4. Statistical Analysis

Computation of percentages was done using Microsoft Excel computer package. The GenStat 15th edition statistical package was used for analysis of variance (ANOVA) and determinations of correlations. Separation of means was done using the least significant difference (LSD) at* P* < 0.05 for year one data whereas Tukey's honest significant difference (HSD) test was used for the year two data at* P* < 0.05. Data for each of the sites were analysed separately as the cropping system effect was the only emphasized factor for year one whereas previous cropping system and N fertilizer application were the two factors considered for year two. However, having two sites served a function of increased repeatability of the experiment.

## 3. Results

### 3.1. The VAM Fungal Colonisation in Pigeon Pea, Cowpea, and Maize Roots as Influenced by Different Cropping Systems at the Lilongwe and Dowa Sites

Results show that there were no significant differences (*P* < 0.05) in percent root length colonised by the VAM fungi in all the three crops as influenced by the cropping systems (Table 1). However, looking across all the crops, maize had the lowest values of percent root length colonised by VAM fungi at both sites.

### 3.2. The VAM Fungal Colonisation of Maize Roots as Influenced the Previous Cropping Systems and N Application at the Lilongwe Site

**Figure 2:**
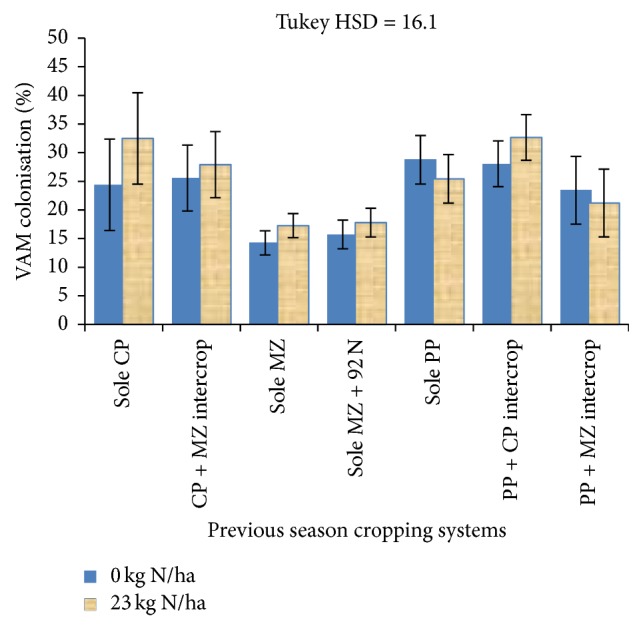
VAM colonisation as influenced by previous cropping systems and N fertilizer application for Lilongwe site.* Key*. Each error bar represents a standard error of the mean; CP = cowpea; MZ = maize; PP = pigeon pea; 92N = 92 kg N ha^−1^.

Results show that there were signification differences (*P* < 0.05) in percent of VAM fungal colonisation in rotational maize roots as affected by the previous season's cropping systems (Figure 2). All treatments that involved legumes (legume-based) in the previous season, that is, pigeon pea and cowpea, grown as sole crops or legume-cereal or legume-legume intercrops, showed significantly higher (*P* < 0.05) percent colonisation of maize roots by VAM fungi ranging from 39% (in previous PP + MZ) to 50% (in previous sole PP) than VAM fungal colonisation in maize not supplied with N fertilizer that followed sole maize. Similarly, the previous season legume-based systems led to significantly higher VAM fungal colonisation, by the range of 19% (in previous PP + MZ) to 47% (in previous PP + CP) in maize supplied with 23 kg N ha^−1^ that was preceded by sole maize of the previous season.

The supplemental 23 kg N ha^−1^ to the rotational maize seemed to have a slight positive influence on VAM fungal colonisation levels, though no significant differences were obtained. It showed nonstatistically different results with the pigeon pea plus maize intercrop effects of VAM fungal colonisation on rotational maize. On the other hand, the 92 kg N ha^−1^ applied to one control plot in the previous season did not show significant effect on VAM fungal colonisation of rotational maize. No significant interaction was observed between previous cropping systems and the 23 kg N ha^−1^ applied to the rotational maize.

### 3.3. The VAM Fungal Colonisation of Maize Roots as Influenced by the Previous Cropping Systems and N Application at the Dowa Site

**Figure 3:**
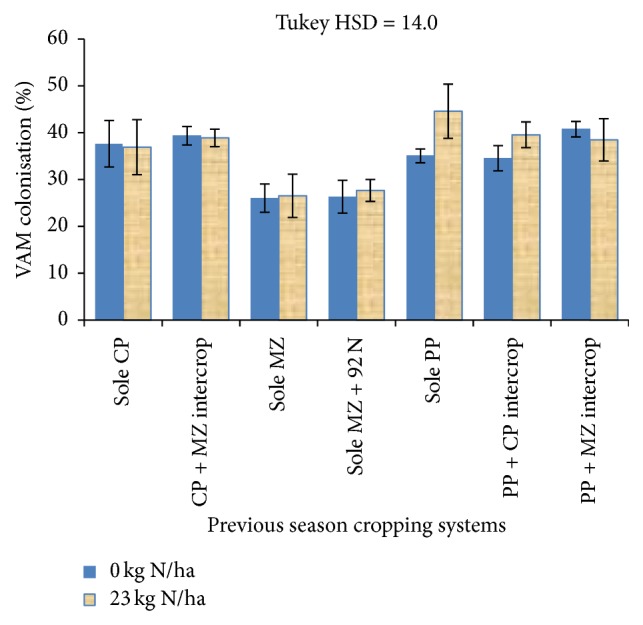
VAM colonisation of maize roots as influenced by previous cropping systems and N fertilizer application for Dowa site.* Key*. Each error bar represents a standard error of the mean; CP = cowpea; MZ = maize; PP = pigeon pea; 92N = 92 kg N ha^−1^.

Results for the Dowa site VAM fungal percent colonisation as influenced by the previous season cropping systems are shown in Figure 3. Similar to the Lilongwe site, results show that there were signification differences (*P* < 0.05) in percent root colonisation as affected by the previous season cropping systems. All treatments that involved legume-based cropping systems in the previous season showed significantly higher (*P* < 0.05) percent colonisation of maize roots by VAM fungi, ranging from 15% (in previous PP + CP) to 36% (in previous PP + MZ) than VAM fungal colonisation in the maize at zero N fertilizer application that followed previous season sole maize. Similarly, where maize was supplied with 23 kg N per ha^−1^, the previous season legume-based systems led to significantly higher VAM fungal colonisation by the range of 28% (in previous sole CP) to 40% (in previous sole PP) in maize roots but the application of N fertilizer did not significantly affect VAM fungal colonisation levels, though they showed some slight increases. In both cases there were no significant interaction between the previous cropping system and 23 kg N ha^−1^ fertilizer application.

### 3.4. Pearson Correlations between Colonisation Percentage and Plant Tissue P Concentration, P Uptake, Total Dry Matter Yields, and Other Parameters for Year One

The Pearson correlation coefficients were determined to assess the association between the VAM fungal colonisation percentage of roots of crops in year one and the plant tissue P concentration (% P), P uptake, total dry matter yields, BNF/plant, nodule number, and nodule dry weights. Results (Table 2) show positive correlation coefficients for all parameters but mostly with nonsignificant* P* values with the exception of P content in maize which showed a significant correlation with level of VAM fungal colonisation (*r* = 0.659,* P* < 0.02) and a few others with* P* values approaching significant levels (from slightly above 0.05 to 0.1).

### 3.5. Pearson Correlations between VAM Fungal Colonisation Percentage and Maize Plant Tissue P and N Concentrations, Total Dry Matter, and Grain Yields for Year Two

Pearson correlations for year two data similarly showed positive associations between VAM fungal colonisation of maize roots and parameters such as P and N concentrations, total dry matter, and grain yields (Table 3). Results showed significant *r* values (*P* < 0.05) for VAM fungal colonisation versus grain yield for Lilongwe site and VAM fungal colonisation versus TDM and grain yields for the Dowa site, under treatments that received inorganic N fertilizer.

## 4. Discussion

Elucidation of levels of VAM fungal colonisation in predominant cropping systems in Malawi such as sole cropping, cereal-legume, and legume-legume intercrops, can be of importance in the development of sustainable agricultural systems. Results in this study showed that the degree of colonisation of each crop that is pigeon pea, cowpea, and maize by VAM was not affected by the cropping system. However, maize, compared with the other crops, showed relatively low VAM colonisation levels. These observations show both similarities and differences to some studies on VAM colonisation in intercropping systems [40]. The nonsignificant differences due to cropping systems can be attributed to the type of crop species involved in the present intercropping study and probably the selectivity of unexplored mycorrhizal fungal species in the soils at the study sites. In a study involving sole maize and maize intercropped with agroforestry species of* Sesbania* and* Gliricidia* in Southern Malawi, on the frequency of occurrence of VAM fungal species, Jefwa [30] reported that out of the 12 VAM fungal species identified, five species occurred most in the maize monocrop whereas the remaining seven were not affected by the cropping systems.

On the other hand, Hage-Ahmed et al. [21] reported three scenarios which showed significant increases and decreases and nonsignificant differences from an intercropping study involving tomato, where VAM fungal colonisation of tomato was increased in an intercrop with leek, while no significant differences were observed when intercropped with cucumber and basil but decreased when intercropped with fennel. These observations were attributed to differences in the establishment of symbioses as affected by different root sizes that in turn affect their influence in the soil ecosystem, but also to the effect of VAM species on plant competitions. In the current study, all the three crops are mycorrhizal as reported in many studies [41–43] and, therefore, are very unlikely to cause suppressive effects on the intercropping partners. However, since no additional evaluation of VAM fungal species was undertaken in this study, therefore, comprehensive evaluation of the intensities of VAM fungal colonisation was not possible.

However, in the second season there were significantly higher colonisation levels of maize roots by the VAM fungi as influenced by the previous season legume-based cropping systems of sole pigeon pea, sole cowpea, and their legume-legume and maize-legume intercrops than in the maize following maize rotational system. This observation is consistent with a number of similar studies. In a study conducted in Zimbabwe, Lekberg et al. [29] reported slightly higher VAM fungal colonisation in maize grown after lablab and pigeon pea than VAM fungal colonisation in maize grown after maize rotational system. On the other hand, Bagayoko et al. [44], in a study done in Niger, reported 45% native VAM fungal colonisation of pearl millet roots when pearl millet followed cowpea in rotation whereas 27% VAM fungal colonisation was observed on pearl millet roots when it followed another pearl millet in rotation. Arihara and Karasawa [41] reported increased mycorrhizal colonisation and yield components in rotational maize after mycorrhizal crops, that is, soybean, sunflower, maize, and potato as compared to maize after nonmycorrhizal crops, rape, and sugar beet. On the other hand, higher positive correlations of maize yield components with VAM fungal colonisation when maize was grown after lablab and pigeon pea were also reported after both legumes in Zimbabwe [29] and after pigeon pea in Nigeria [43]. Furthermore, Njeru et al. [45] reported increased indigenous VAM fungal colonisation of rotational maize roots after cover crop legumes such hairy vetch* (Vicia villosa)*, common pea* (Pisum sativum)*, and broad bean* (Vicia faba)* which was attributed to the higher ability of some crop species to sustain VAM fungal natural communities more than others. Furthermore, in a study on comparison of the effects of legume (lupine) residues and nonlegume (wheat) residues on VAM fungal proliferation, higher mycorrhizal fungi colonisation was noted in treatments applied with lupine which was attributed to the higher nutritional content in the legume residues which boosted mycorrhizal development [46]. Therefore, the VAM fungal sustainability and residue quality effects of the legumes can be main factors that increased VAM fungal colonisation in the rotational maize of the present study.

The Pearson correlation coefficients were computed to assess the associations between the VAM fungal colonisation of pigeon pea, cowpea, and maize roots and various parameters including plant P, P uptake, content, BNF, nodule number, and nodule dry weights (Table 2) for the first season. Results showed positive associations between VAM fungal colonisation of roots of plants under study and their plant tissue P, total P uptake, total dry matter yield, BNF, nodule numbers, and nodule dry weight but mostly showed nonsignificant* P* values, except for phosphorus content in maize which had a significant correlation. The weak association and nonsignificant correlation coefficients predominant in this study could be a contrast to the advantages of VAM fungal colonisation as reported in many studies [11, 14, 43]. However, VAM studies lead to a number of uncommon observations in terms of associations with plant P contents and even effects due to available soil P which was relatively high in this study [47, 48]. Furthermore, both levels of soil P and levels of VAM colonisation could be the reasons for observations in this study. Though some authors have considered 40% as high colonisation, but based on meta-analysis data of 91 laboratory and field based studies Treseder [49] reports that in most cases response ratio of plant biomass and plant P concentration increases as percent root length colonised (PRLC) increases and the benefit of PRLC becomes distinctly prominent if PRLC reaches 60% or more. Therefore, the VAM root length colonisation levels that never reached 60% and rarely exceeded 40% in this study could be the main factor contributing to the weak positive associations observed between VAM fungal colonisation and parameters such as plant P content, BNF, and dry matter yields.

However, for year two data, significant correlations were observed between VAM fungal colonisation and total dry matter and grain yields especially in treatments that were fertilized with inorganic N fertilizer. This result may be a contradiction to number of other studies that suggest that VAM colonisation is suppressed with addition of inorganic fertilizers such as N and P [50–52]. On the contrary, the results are in agreement with a study that shows increase in mycorrhizal colonisation with fertilization by N or P in nutrient limited soils, varying with species, with incidences of* Glomus* spp. increasing in relatively fertile soils [53]. Although no fungal species identification was conducted in this study, it should be noted that, in this study, the soils showed very low N (Section 2.1) levels. Therefore, addition of readily available mineral N would similarly lead to positive interactive effects on the maize yields, though analysis of variance only showed slight increases in colonisation as influenced by N application (Figures 2 and 3). From what was observed in this study, further investigations on thresholds at which nutrient additions affect VAM development negatively or positively are needed.

## 5. Conclusions

From this study, it can be concluded that VAM fungal colonisation was not affected by the legume-based cropping systems such as sole cropping, cereal-legume, and legume-legume intercrops involving pigeon pea, cowpea, and maize. On the other hand, all the legume-based cropping systems showed significant positive effect on VAM fungal colonisation of the subsequent maize grown in short rotation. Furthermore, there were positive correlations between plant roots' VAM fungal colonisation and the plant P content, nodule numbers, BNF, and total dry matter yields in year one. Similarly, positive correlations between VAM fungal colonisation and maize yields were also noted in year two. Therefore, integrating diversified legume-based cropping systems can be a good approach in promoting VAM fungal proliferation that contributes to increasing plant P uptake, which also has positive effects on BNF, crop growth, and yields. Furthermore, the increased P acquisition and BNF are among the key components of soil health improvement for sustainable agriculture production, in most soils of sub-Saharan Africa. Additionally, more research needs to be done to understand the interactions between cropping systems and existing VAM fungal species, their abundance, and diversity on Malawi soils. Isolation of more adapted species for inoculant production can be another good step forward in alleviating soil health problems. Furthermore, studies are also needed to establish thresholds at which addition of nutrients such as N affects VAM fungal development positively or negatively.

## Figures and Tables

**Table 1:** Effect of cropping system on VAM fungal colonisation on pigeon pea, cowpea, and maize at the two study sites in year one (2013/14 cropping season).

Crop	Cropping system	Lilongwe	Dowa
		VAM colonisation (%)	VAM colonisation (%)
PP	Sole PP	25.0	41.1
	PP + CP intercrop	26.9	36.9
	PP + MZ intercrop	26.1	37.4
	LSD	11.6^ns^	10.1^ns^
	F pr.	0.91	0.51
	CV%	19.8	11.6

CP	Sole CP	33.2	46.7
	CP + PP intercrop	28.2	40.1
	CP + MZ intercrop	25.5	41.2
	LSD	11.0^ns^	20.5^ns^
	F pr.	0.261	0.660
	CV%	16.8	21.2

MZ	Sole MZ	15.2	24.6
	MZ + PP intercrop	17.3	22.6
	MZ + CP intercrop	14.5	20.8
	Sole MZ + 92N	19.4	23.4
	LSD	15.9^ns^	4.4^ns^
	F pr.	0.872	0.300
	CV%	47.8	9.6

*Note*. LSD = least significant difference at 5%; F pr. = F probability; CV% = coefficient of variation; CP = cowpea; MZ = maize; PP = pigeon pea; 92N = 92 kg N ha^−1^; ns = nonsignificant.

**Table 2:** Pearson correlations between colonisation percentages and plant tissue P concentration, P uptake, total dry matter yields, BNF/plant, nodule number, and nodule dry weights for year one.

Crop	VAM colonisation (%) and parameters	Lilongwe		Dowa	
		*r*	*P* value	*r*	*P* value
PP	% P	0.395	0.293	0.185	0.634
	P uptake	0.349	0.365	0.488	0.182
	TDM	0.170	0.661	0.045	0.909
	BNF	0.191	0.623	0.188	0.627
	Nodule number	0.067	0.861	0.577	0.154
	Nodule dry weight	0.368	0.330	0.470	0.202

CP	% P	0.252	0.513	0.379	0.290
	P-uptake	0.645	0.061	0.557	0.120
	TDM	0.067	0.864	0.660	0.053
	BNF	0.630	0.069	0.479	0.193
	Nodule number	0.155	0.689	0.657	0.055
	Nodule dry weight	0.438	0.238	0.650	0.058

MZ	% P	0.659	0.020^*∗*^	0.558	0.283
	P-uptake	0.455	0.138	0.210	0.352
	TDM	0.378	0.226	0.101	0.521

*Note*. Correlations were only presented for level of VAM fungal colonisation (%) against the other parameters; *∗* = significant at *P* < 0.05; *r* = correlation coefficient; CP = cowpea; MZ = maize; PP = pigeon pea; Nod = nodule; TDM = total dry matter yield.

**Table 3:** Pearson correlations between VAM colonisation percentages and plant tissue P concentration (% P), plant tissue nitrogen concentration (% N), total dry matter, and grain yields for year two.

Treatment	VAM colonisation (%) and parameters	Lilongwe		Dowa	
		*r*	*P* value	*r*	*P* value
MZ + 0N	% P	0.082	0.725	0.139	0.099
	% N	0.302	0.184	0.294	0.196
	TDM	0.204	0.365	0.233	0.309
	Grain	0.386	0.084	0.257	0.264

MZ + 23N	% P	0.300	0.187	0.169	0.747
	% N	0.341	0.130	0.266	0.244
	TDM	0.408	0.067	0.522	0.015^*∗*^
	Grain	0.513	0.018^*∗*^	0.597	0.004^*∗*^

*Note*. Correlations were only presented for level of VAM fungal colonisation (%) against the other parameters; *∗* = significant at *P* < 0.05; *r* = correlation coefficient; MZ = maize; MZ + 0N = Maize with nitrogen fertilizer application; MZ + 23N = maize with 23 kg N ha^−1^ application; TDM = total dry matter yield.

## Abbreviations

AGRA: Alliance for Green Revolution in Africa
ANOVA: Analysis of variance
BNF: Biological nitrogen fixation
CP: Cowpea
Cu: Copper
EPA: Extension Planning Area
H_2_O_2_: Hydrogen peroxide
HSD: Honest significant difference
ISFM: Integrated soil fertility management
K: Potassium
KOH: Potassium hydroxide
LSD: Least significant difference
LUANAR: Lilongwe University of Agriculture and Natural Resources
MZ: Maize
N: Nitrogen
P: Phosphorus
PP: Pigeon pea
PRLC: Percent root length colonised
RCBD: Randomized complete block design
USDA: United States Department of Agriculture
VAM: Vesicular arbuscular mycorrhiza
Zn: Zinc.
